# Correction to: CoQ10 reduces glioblastoma growth and infiltration through proteome remodeling and inhibition of angiogenesis and inflammation

**DOI:** 10.1007/s13402-023-00846-1

**Published:** 2023-08-02

**Authors:** Javier Frontiñán-Rubio, Emilio Llanos-González, Sonia García-Carpintero, Juan Ramón Peinado, Inmaculada Ballesteros-Yáñez, Margarita Villar Rayo, José de la Fuente, Víctor M. Pérez-García, Luis A. Perez-Romasanta, Marcos Malumbres, Francisco J. Alcaín, Mario Durán-Prado

**Affiliations:** 1 Department of Medical Sciences, Faculty of Medicine, University of Castilla-La Mancha, Ciudad Real, 13071 Spain; 2 Oxidative Stress and Neurodegeneration Group, Faculty of Medicine, Regional Centre for Biomedical Research, University of Castilla-La Mancha, Ciudad Real, Spain; 3 EMAS Group, Faculty of Medicine, Regional Centre for Biomedical Research, University of Castilla-La Mancha, Ciudad Real, Spain; 4 SaBio Research Group, Hunting Resources Research Institute (IREC), Ciudad Real, Spain; 5 Laboratory of Mathematical Oncology, University of Castilla-La Mancha, Ciudad Real, Spain; 6 Radiology and Medicinal Physics, Institute for Biomedical Research of Salamanca (IBSAL), Salamanca, Spain; 7 Cell Division and Cancer Group, Spanish National Cancer Research Centre (CNIO), Madrid, Spain

**Cellular Oncology (2022) 46:65-77**

10.1007/s13402-022-00734-0

In this article the following information should have been included in the Funding section: ‘Open access funding was provided by project 2022-GRIN-34247’.

